# Temporal sequence learning in reentrantly coupled winner-take-all networks of spiking neurons

**DOI:** 10.1186/1471-2202-14-S1-P271

**Published:** 2013-07-08

**Authors:** Jeffrey L McKinstry

**Affiliations:** 1 The Neurosciences Institute, La Jolla, California 92037, USA

## 

**Figure 1:**
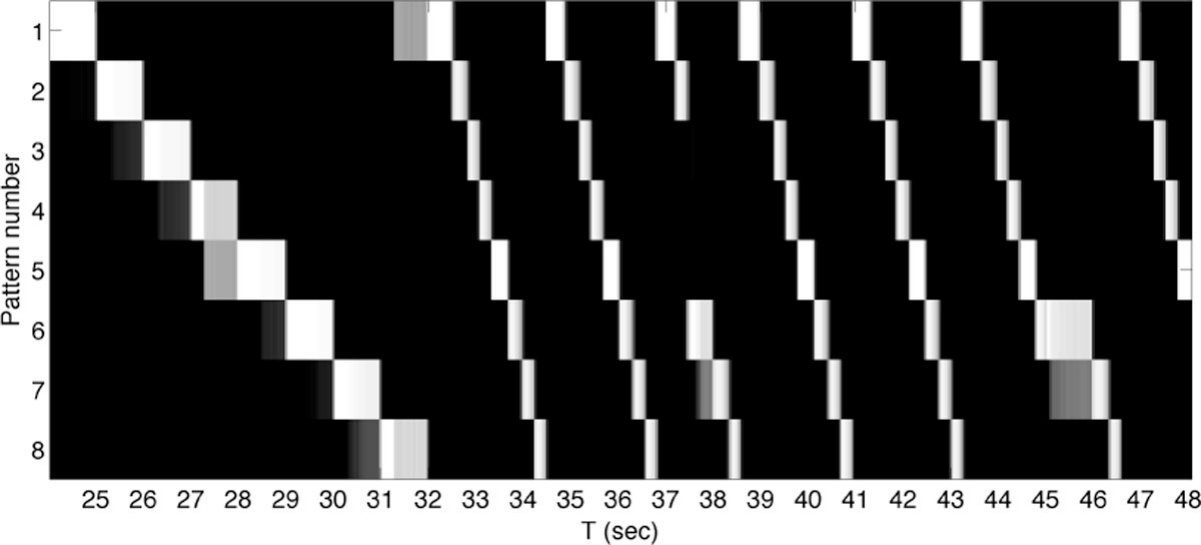
After training, a large-scale network of approximately 4,000 spiking neurons transitions between activity patterns, in the absence of external cues, reflecting the learned sequence

Patterns of activity in brains are commonly composed of temporal sequences of periods with steady-state firing rates lasting several hundred milliseconds separated by sharp transitions during movement [1], perception [2], and remembering [3]. Although network models involving mean-firing-rate neurons have been used to generate sequential neural activity [1], spiking networks with such capability require further exploration. We describe how large-scale Winner-Take-All (WTA) spiking networks can be coupled together and trained to generate such sequential neural activity. These networks are composed of conductance-based excitatory and inhibitory spiking neurons [4]. Model synapses were subject to short-term synaptic plasticity and spike-timing dependent plasticity (STDP). Each network was a Center-Annular-Surround (CAS) network, a variant of center-surround networks that we have found to effectively generate WTA dynamics in large-scale networks of spiking neurons. Two CAS networks were coupled together reentrantly to form a network capable of sequence learning and recall. We found that networks of this sort can be trained to respond to a sequence of sensory cues by generating temporally ordered patterns of neuronal activity. The patterns consist of brief steady states separated by sharp transitions that resemble those observed experimentally. After training, synaptic changes resulting from STDP acting on connections between the coupled networks formed a link between temporally adjacent patterns of neural activity within the sequence. Figure 1 reflects an analysis of spiking data from this simulated network trained with a repeating sequence of eight cues. Each row in the figure plots the match score over time to one of the eight patterns of neural activity corresponding to one of the cues. White is a perfect match, while black indicates a complete mismatch. The last training repeat is from t = 24 to t = 32. Subsequently, external cues were removed and network activity continued, cycling through all eight patterns until another input cue was presented. Then the activity pattern changed to the activity pattern appropriate to the cue presented (see at t = 37), continuing the sequence when the cue was removed. The system was robust with respect to various initial conditions.

We also used the present model to control specific motor sequences in a robotic device. The population activity pattern in this modeled nervous system has similarities to that observed in primate frontal cortex during multi-segmented limb movements.
